# A New Medical Dictionary

**Published:** 1890-05

**Authors:** 


### A New Medical Dictionary.

There has just been issued from the publishing house of P. Blakiston, Son & Co., of Philadelphia, a new medical dictionary, by George M. Gould, Ophthalmic Surgeon of the Philadelphia Hospital, Clinical Chief Ophthalmological Department, German Hospital, Philadelphia.

This work includes all the words and phrases used in medicine with their proper pronunciation and definitions, with elaborate tables of the bacilli, micrococci, leucomaines, ptomaines, etc.; of the arteries, ganglia, muscles, nerves, and plexuses; of weights and measures, thermometers, etc.; and appendices containing tables with analysis of the waters of the mineral springs of the United States, and tables of vital statistics. The work has been prepared *De Novo.* and seems to well cover the ground of medical nomenclature up to the present day. As is well known since the preparation of medical dictionaries prior to three to five years ago many terms and names are not found, because they have been either introduced or established in use within that time. All such words are brought into this work. The definitions in this work of all names, terms and words are made very clear and understandable. In a cursory glance through the work we have not been able to find that any word or name now in common use has been omitted.

This work has the advangage of being comprehensive, very complete indeed, and yet not made voluminous by unnecessarily extended definitions, explanations and descriptions. It contains all in these respects that the student or practitioner really needs. It includes several thousand new words not contained in any other book of its size, and until the present year not in any other medical dictionary. It is of convenient size and form and the price moderate, $3.25.

